# Emerging role of N-acetyltransferase 10 in diseases: RNA ac4C modification and beyond

**DOI:** 10.1186/s43556-025-00286-3

**Published:** 2025-07-01

**Authors:** Lin Jiao, Yanjun Si, Yushan Yuan, Xinxing Lei, Qian Jiang, Lijun Yang, Wenhao Mao, Binwu Ying, Liwei Ma, Ting Sun

**Affiliations:** 1 Department of Laboratory Medicine, West China Hospital, Sichuan University, Chengdu, 610041 China; 2 Department of Laboratory Medicine, Shenzhen People’s Hospital (The Second Clinical Medical College, Jinan University; The First Affiliated Hospital, Southern University of Science and Technology), Shenzhen, Guangdong 518020 China; 3 Department of Clinical Laboratory, Key Clinical Laboratory of Henan Province, The First Affiliated Hospital of Zhengzhou University, Zhengzhou, 450052 China; 4 Department of Oncology, The First Affiliated Hospital of Zhengzhou University, Zhengzhou, 450052 China

**Keywords:** N-acetyltransferase 10, RNA modification, N4-acetylcytidine, Cancer, Disease

## Abstract

N^4^-acetylcytidine (ac4C), a conserved RNA modification, plays critical roles in RNA stability and translation. As the primary enzyme catalyzing ac4C, N-acetyltransferase 10 (NAT10) is increasingly implicated in diverse diseases. This review systematically explores NAT10’s multifaceted contributions to cancer, autoimmune disorders, infectious diseases, cardiovascular conditions, and metabolic syndromes. In cancer, NAT10 drives malignancy by enhancing oncogenic processes such as proliferation, metastasis, and therapy resistance, with overexpression linked to poor prognosis across multiple malignancies. Beyond oncology, NAT10 dysregulation is associated with autoimmune diseases like rheumatoid arthritis and systemic lupus erythematosus, where it modulates immune responses through RNA acetylation. In infectious contexts, NAT10 influences sepsis progression and viral pathogenesis by stabilizing pathogen-related RNAs, while in cardiovascular diseases, it exacerbates myocardial injury and heart failure through ac4C-dependent and independent pathways. Additionally, NAT10 promotes metabolic dysfunction-associated steatotic liver disease by regulating lipid metabolism genes. The review further discusses therapeutic strategies targeting NAT10, including small-molecule inhibitors and gene silencing approaches, which show promise in preclinical models by suppressing tumor growth, enhancing chemosensitivity, and mitigating inflammatory damage. By integrating molecular insights and clinical relevance, this work underscores NAT10 as a pivotal regulator of disease mechanisms and a potential target for future therapeutic interventions. Future research should address context-dependent roles, refine ac4C detection methods, and explore combinatorial therapies to overcome resistance mechanisms.

## Introduction

Epigenetic modifications refer to cellular protein, DNA or RNA decorated with diverse chemical modifications like methylation, acetylation, which participate in all aspect of genetics metabolism [1–4]. These modifications, which are reversible and heritable, play a crucial role in regulating gene expression and influencing disease progression without altering DNA sequences [5, 6]. Among these, RNA modifications have emerged as critical regulators of gene expression, expanding the functional diversity of RNA beyond its canonical roles in transcription and translation [7–10]. Over 100 distinct RNA modifications have been identified, with N^4^-acetylcytidine (ac4C) standing out as a unique and evolutionarily conserved epitranscriptomic mark [11, 12]. Initially discovered in bacterial tRNA and rRNA, ac4C was later identified in eukaryotic mRNA and non-coding RNAs (ncRNAs), where it enhances RNA stability, translation efficiency, and transcriptional fidelity [13, 14].

The ac4C modification in eukaryotes is specifically catalyzed by N-acetyltransferase 10 (NAT10), the only characterized enzyme demonstrated to perform this acetylation process [15]. Structurally, NAT10 contains a catalytic acetyltransferase domain coupled with RNA-binding motifs, enabling it to acetylate cytidine residues in RNA substrates, including tRNA, rRNA, mRNA, and long non-coding RNAs (lncRNAs) [16, 17]. Beyond its enzymatic role, NAT10 participates in diverse biological processes, such as aging [18–21] and cell division [22–26], underscoring its multifaceted regulatory potential. Recent studies have highlighted NAT10’s involvement in pathological conditions, particularly cancer, where its dysregulation promotes tumor proliferation, metastasis, and therapy resistance by stabilizing oncogenic transcripts [27, 28]. Despite these advances, the precise mechanisms linking NAT10-mediated ac4C modifications to disease progression remain incompletely understood, necessitating a comprehensive synthesis of current knowledge.

This review aims to elucidate the emerging roles of NAT10 and ac4C modification in health and disease. We first outline the historical discovery of ac4C and the functional insights into NAT10. Subsequently, we systematically analyze NAT10’s regulatory roles in various diseases and the mechanisms in pathogenesis, diagnosis, prevention, and treatment of diseases. Furthermore, we explore the therapeutic potential of targeting NAT10, highlighting its utility as a prognostic biomarker and a druggable node in disease treatment. By integrating recent advances and unresolved questions, this review provides a roadmap for future research, highlighting the critical importance of elucidating NAT10-driven ac4C modifications in disease pathogenesis and their potential as therapeutic targets.

## NAT10: structure, function, and regulation

### Biochemical properties of NAT10

**Fig. 1:**
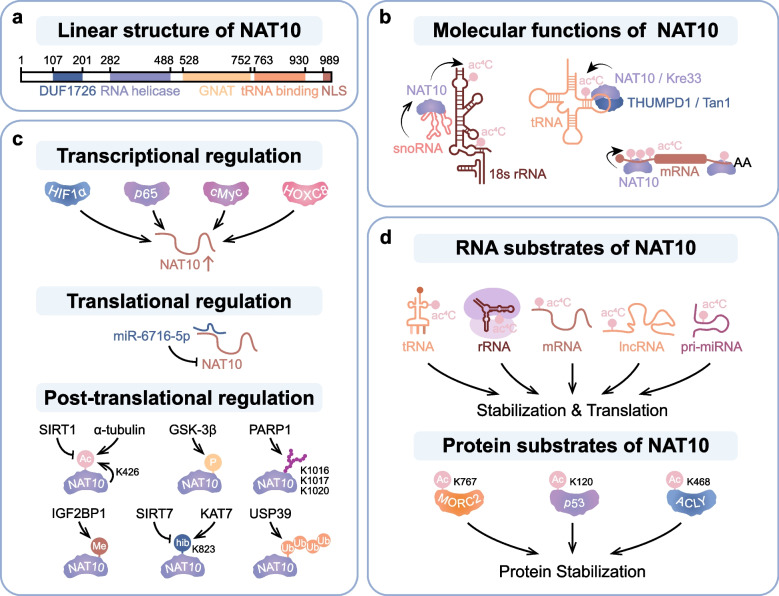
NAT10: structure, catalytic function, regulation, and substrate recognition. **a** Linear structure of NAT10. **b** NAT10-mediated ac4C modification in mRNA, tRNA, and 18 s rRNA. **c** Upstream regulation of NAT10 at the level of transcription, translation and post-translation. **d** The RNA substrates and protein substrates of NAT10

NAT10, a key component of the N-acetyltransferase family, is an abundant nucleolar protein with both lysine and RNA cytidine acetyltransferase activities. NAT10 is highly conserved among different species, from yeast to humans, and the structure of NAT10 gene shares a domain architecture consisting of RNA helicase linked to a GNAT domain [15, 29–32]. Human NAT10 contains nucleolar localization sequence (NLS) at their C-terminal end, which dictates its subcellular localization (Fig. 1). NAT10 is ubiquitously expressed in diverse tissues, encompassing lymphoid tissues, kidneys, livers, cerebral cortex, and the central nervous system [33]. In physiology, NAT10 is mainly involved in regulating telomerase length [19], nuclear protein [18, 34], nuclear shape [20] and cell division [35–37].

### Substrates and targets of NAT10 acetylation

The first identified homolog of the NAT10 enzyme, which is involved in RNA modification, is TmcA in Escherichia coli [38] and Rra1/Kre33 in yeast [39]. Initially, Cytidine acetyltransferase (TmcA) catalyzed the *E.Coli* elongator tRNA^met^ into ac4C, ensuring the precise recognition of the AUG codon by preventing misreading of near-cognate AUA codon. Kre33, the yeast homolog of NAT10, interacts with the conserved adaptor TAN 1 to catalyze ac4C modification of yeast tRNA and 18S rRNA, thereby promoting correct translation [39, 40]. In S. cerevisiae, rRNA cytidyl acetyltransferase 1 (Rra1) catalyzes the formation of ac4C at position 1773 in the 18 S rRNA using both acetyl-CoA and ATP as substrates. In Human, NAT10 catalyze ac4C modification with broad specificity ranging from histones and microtubules to tRNA and rRNA.

In addition to RNA acetyltransferase activity, NAT10 is also involved in multiple cellular processes by acting as a lysine acetyltransferase [41]. Belonging to the Gcn5-related N-acetyltransferase (GNAT) family of lysine acetyltransferases (KATs), NAT10 was first identified as a histone acetyltransferase facilitating transcriptional coactivation of the telomerase gene [42]. As a protein lysine acetyltransferase, NAT10 is indispensable in the process for transferring the acetyl groups to histone and non-histone proteins [43, 44]. Collectively, NAT10 exhibits dual functionality as both an RNA and protein acetyltransferase, bridging RNA quality control in translation with epigenetic and post-translational regulation (Fig. 1). Its evolutionary conservation across species underscores its fundamental role in maintaining cellular homeostasis through diverse acetylation-dependent mechanisms.

### Regulation of NAT10 activity and stability

#### Transcriptional regulation

The expression of NAT10 could be regulated by transcription factors like HIF1α, p65, c-myc and HOXC8. HIF1α is a crucial transcriptional factor and plays an important role in cellular response to hypoxia [45]. Under hypoxia circumstances, HIF1α mediated the transcriptional upregulation of NAT10 and subsequent glucose metabolism reprogramming by mediating SEPT9 mRNA ac4C modification [46]. Central to this upregulation is the activation of the NF-κB signaling cascade, wherein the pivotal NF-κB subunit p65 binds directly to the NAT10 promoter region to stimulate transcription. This interaction bolstered cisplatin chemoresistance in bladder cancer cells by augmenting DNA damage repair mechanisms, underscoring the pivotal role of NAT10 in mediating therapeutic resistance via modulation of cellular repair pathways [47]. Other transcriptional factors like c-myc and HOXC8 also induced NAT10 upregulation. The transcription factor c-myc is activated and upregulates NAT10 expression to facilitate cancer development via cell cycle control [48], and transcription factor HOXC8 activates NAT10 by binding to its promoter and thereby stimulating translation efficiency of FOXP1 eventually leading to increased glycolysis [49].

#### Translational regulation

The translational regulation of NAT10 has been reported to be performed by non-coding RNAs. miR-6716-5p acted as a crucial regulator of NAT10 by binding to the 3’- untranslated regions (UTRs) of NAT10 mRNA and inhibiting NAT10 expression. Through inhibiting NAT10 expression, miR-6716-5p downregulated E-cadherin levels and promoted colorectal cancer cells migration and invasion [50].

#### Posttranslational regulation

Merging evidence underscores the pivotal role of post-translational modifications (PTMs) in modulating NAT10’s expression, encompassing acetylation, phosphorylation, methylation, poly(ADP-ribosyl)ation (PARylation), and 2-hydroxyisobutyrylation (Khib). Within the realm of acetylation modifications, as reported by Yan et al., NAT10 undergoes acetylation by α-tubulin acetyltransferase 1 (ATAT1), enabling it to perform its RNA acetyltransferase function by enhancing the ac4C level in tRNASer-CGA-1–1 [51]. As reported by Cai et al., NAT10 also undergoes self-acetylation at residue K426, essential for its efficacy in rRNA transcription activation [41]. Additionally, Liu et al. unveiled SIRT1’s role in deacetylating NAT10 during energy scarcity, facilitating the shift from rRNA biosynthesis to autophagy by abolishing NAT10-mediated transcriptional repression of Che-1 [52]. For phosphorylation modification, NAT10 was phosphorylated by GSK-3β, which is a versatile serine/threonine kinase that regulates the breakdown of target proteins. GSK-3β influences NAT10 stability through the phosphorylation coupled ubiquitin/proteasome pathway [53]. Moreover, PARP1, a highly conserved DNA damage-dependent enzyme, mediates the PARylation of NAT10 [54]. Upon DNA injury, PARP1-catalyzed PARylation of NAT10 at specific lysine sites (K1016, K1017, K1020) is pivotal for its nuclear relocation and subsequent DNA damage mitigation [55]. Furthermore, NAT10 mRNA levels are influenced by IGF2BP1 in an m6 A-regulated manner, steering ac4C modifications in ACOT7 mRNA and impacting fatty acid metabolism. Lastly, NAT10 serves as a substrate for Khib modification, where K823 modification of NAT10 enhances its association with USP39, promoting NOTCH3 mRNA stabilization and consequently fostering esophageal tumor dissemination [56].

Overall, these studies demonstrate that NAT10 can be regulated by a range of transcription factors, non-coding RNAs and posttranslational modifications (Fig. 1). Investigation of the upstream regulators of NAT10 helps us to better understand the mechanism of NAT10 in cancer and provides potential targets for cancer therapy.

## ac4C RNA modification via NAT10

The discovery of ac4C as a dynamic regulator across RNA species has positioned NAT10 as a central player in epitranscriptomic studies. As the sole identified"writer"enzyme for ac4C, NAT10 orchestrates site-specific cytidine acetylation in diverse RNA substrates, including mRNA, tRNA, rRNA, and non-coding RNAs. This enzymatic process not only fine-tunes RNA structure and function but also bridges acetyl-CoA metabolism with translational control. Below, we dissect the molecular architecture and catalytic logic of NAT10-mediated ac4C installation, a prerequisite for understanding its biological impact.

### Biochemical basis of ac4C catalyzed by NAT10

ac4C is recognized as a universally conserved RNA modification present in prokaryotic and eukaryotic species. Since first identified in yeast tRNA in 1966 [57], ac4C was subsequently reported present in eukaryotic leucine and serine tRNAs [58, 59], as well as in 18S ribosomal RNA (rRNA) [15, 39]. In 2018, Arango and colleagues initially unveiled the ac4C modification on mRNA and their findings suggest that this ac4C modification on mRNA acts as a positive regulator for RNA stability and translation efficiency [60]. They also elucidated that the impact of ac4C on mRNA translation is contingent on its position. In certain scenarios, ac4C in the 5′-UTR of transcripts facilitates upstream initiation while simultaneously inhibiting the use of conventional start codons [31, 61]. When ac4C is situated within mRNA coding sequences, coding DNA sequence (CDS) ac4C thus enhances translation elongation through improving interactions with cognate tRNAs, thereby inhibiting co-translational decay pathways that would otherwise occur at non-optimal codon contexts [60]. Although most ac4C modifications occur in the 5'UTR of transcripts, recent reports have also found that ac4C modifications can occur in the 3'untranslated region of transcript, which is also essential for its translation and stability [62].

As an RNA cytidine acetyltransferase, NAT10 is an only-known ac4C “writer”, which catalyzed ac4C modification of mRNA, tRNA, and 18S rRNA. NAT10 does not work alone, it needs acetyl-coenzyme A (CoA) for acetyl generation and ATP for energy production [38]. NAT10 performs the function through the ATP-dependent manner by transferring an acetyl group from acetyl-CoA to the exocyclic N4-amine of cytidine, catalyzing the formation of ac4C modifications in a variety of RNA substrates. By mass-spectrometric analysis, THUMPD1 in humans and Tan1 in yeast are reported as adapters for acetylation of tRNAs [30]. SNORD13 in humans and U13 C/D box small nucleolar RNA (snoRNA) in yeast are reported as adapters for acetylation of 18S rRNAs [63, 64], a process contingent upon the formation of extensive base-pairing interactions centered around the target cytosine residues, similar to pseudour-idylation pocket formation by the H/ACA snoRNPs. For ac4C modification, NAT10 is the acknowledged ac4C “writer”, however, it is unclear whether there is an “eraser” protein for ac4C modification.

### Metabolism and translational regulation by ac4C

The presence of ac4C on tRNAs contributes to enhancing the high fidelity of protein translation and sustaining the thermotolerance of the organism [65, 66]. The occurrence of ac4C on rRNA is a hallmark feature of thermophilic organisms, playing a crucial role in maintaining the structural stability of the ribosome under high temperatures while also supporting the accuracy of protein translation. The existence of ac4C on mRNAs boosts mRNA stability, protecting it from degradation, and elevates protein translation efficiency. This 5'UTR modification promotes initiation at upstream sequences, competitively inhibiting annotated start codons [67], and can alter the secondary structure of the mRNA, making it more accessible to ribosomes and other translational machinery, hence facilitating efficient and rapid protein production.

In recent years, ac4C modification has been reported to occur not only on mRNA, tRNA, and rRNA, but also on other non-coding RNAs. lncRNA are the primary substrates. It is reported that ac4C modifications of lncRNA SIMALR and CTC-490G23.2 leads to the stabilization and overexpression of lncRNA which promotes cancer malignance [68, 69]. A cluster of lncRNA were also reported hyperacetylated in Alzheimer’s disease, which were associated with the occurrence and development of Alzheimer’s disease [70]. Besides lncRNA, pri-miRNA is also reported as the substrate of ac4C modification. Ac4C of pri-miRNA promotes the processing of pri-miRNA into precursor miRNA which is crucial for cancer initiation and progression [71]. However, there is no mechanism to explain how ac4C modification affects lncRNA expression and miRNA maturation.

Overall, ac4C serves as a vital RNA modification that optimizes various aspects of gene expression, especially in organisms adapted to extreme thermal environments.

## Non-ac4C-dependent functions of NAT10

Besides the classical pathways of regulating RNA stability and translation through mRNA ac4C modification, NAT10 plays major roles in lysine acetylation of histone and non-histone proteins. NAT10 possesses lysine acetylation activity and is implicated in the regulation of DNA damage and chemoresistance. Based on the available researches, it emerges that subnuclear localization of NAT10-specifically whether it resides in the nucleolus versus the nucleoplasm-determines cellular progression outcomes upon the presence or absence of DNA damage in breast cancer. Following DNA damage, NAT10 translocates from nucleolus to nucleoplasm, where it actively participates in pathways including cell cycle arrest, thus playing a pivotal role in maintaining genome stability and tumor survival. The mechanism is specifically manifested in the lysine acetyltranferase ability of NAT10 in regulating MORC2 activities. After DNA damage, the PARylation of NAT10 by PARP1 activation at three conserved lysine residues (K1016, K1017, and K1020) promotes the translocation of NAT10 from the nucleolus to the nucleoplasm. NAT10’s translocation increases its co-localization and interaction with its substrate, MORC2, thereby enhancing the NAT10-mediated MORC2 K767 acetylation. Acetylation of MORC2 is indispensable for triggering the G2 checkpoint, thereby promoting cell survival under DNA-damaging conditions [55, 72]. NAT10 contributes to genome stability by stabilizing the p53 protein. NAT10 acetylates p53 at K120 and enhances its stability through counteracting Mdm2, a process mediated by NAT10's intrinsic E3 ligase activity that promotes Mdm2 ubiquitination and degradation. Following DNA damage, NAT10 translocates to the nucleoplasm and activates p53-dependent cell cycle arrest and apoptosis [73]. Notably, NAT10 expression is significantly downregulated in human colorectal carcinomas, and its overexpression suppresses tumor cell proliferation. These results indicate that targeting NAT10 may enhance cancer cell susceptibility to DNA-damage-inducing chemotherapeutic agents. Moreover, the upregulation of NAT10 was observed in chemo-resistant hepatocellular carcinoma. Upon chemotherapy, NAT10 translocates from the nucleolus to the nucleus, acetylating ACLY at K468 to increase nuclear acetyl-CoA production and stabilize ACLY and promoting chemoresistance [74]. Collectively, NAT10 exhibits multifaceted, non-ac4C-dependent roles in regulating genome stability, DNA damage response, and chemoresistance through substrate-specific acetylation, positioning it as a context-dependent therapeutic target in cancer.

## NAT10 in human diseases: an integrated perspective

NAT10-mediated ac4C RNA modification has emerged as a pivotal regulatory axis in the pathogenesis of diverse human diseases (Table 1). By orchestrating RNA stability, translation efficiency, and non-coding RNA functions, NAT10-driven ac4C dysregulation intersects with multiple pathological hallmarks, including uncontrolled proliferation, immune evasion, metabolic reprogramming, and therapeutic resistance. This section systematically dissects the multifaceted roles of NAT10 and ac4C across cancer biology, autoimmune disorders, infectious diseases, and other clinical contexts. We highlight how aberrant NAT10 expression or ac4C deposition reshapes disease trajectories through molecular mechanisms spanning epigenetic reprogramming, microenvironment remodeling, and stress adaptation. Furthermore, we explore the translational potential of targeting NAT10 as a diagnostic biomarker and therapeutic vulnerability in precision medicine.

**Table 1:** The role of NAT10 in various pathological processes besides cancer

Pathological process	Role	Downstream gene	Molecular mechanism	Function	Ref
Myocardial Infarction	ac4C acetylation	Mybbp1a	NAT10 facilitates the ac4C acetylation of Mybbp1a, increasing its stability, which further triggering p53 activation and suppressing the transcription of SLC7 A11 gene	NAT10 causes cardiomyocyte ferroptosis and exacerbates cardiac ischemia–reperfusion injury	[75]
	ac4C acetylation	Tfec	piRNA targets NAT10-mediated ac4C of Tfec mRNA, which further upregulates Bik	NAT10 promotes the myocardial injury caused by cardiomyocyte apoptosis in ischemia heart diseases	[76]
	ac4C acetylation	EGR3	NAT10 mediates EGR3 mRNA acetylation	NAT10-mediated EGR3 mRNA acetylation promotes myocardial fibrosis after myocardial infarction	[77]
Heart Failure	independent of the ac4C catalyzing activity	Uqcr11, Uqcrb	NAT10 binds to Uqcr11 and Uqcrb respective transcripts, inhibits their mRNA nuclear export, and reduces the translation of their mRNA	NAT10 promotes mouse cardiac regeneration and improves cardiac function after injury	[78]
Rheumatoid Arthritis (RA)	ac4C acetylation	PTX3	NAT10 promotes stability and translation efficiency of N4-acetylated PTX3 mRNA	NAT10 promotes rheumatoid synovial aggression and inflammation	[79]
Systemic Lupus Erythematosus (SLE)	ac4C acetylation	USP18, GPX1, RGL1	ac4C-related transcripts regulate mRNA catabolic processes and translational initiation	NAT10 promotes immune and inflammatory responses of SLE processes	[80]
Ankylosing Spondylitis	-	-	-	NAT10 mRNA together with neutrophil percentages could predictive new-onset ankylosing spondylitis patients	[81]
Sepsis	ac4C acetylation	ULK1	NAT10 promotes ULK1 expression through mRNA acetylation	NAT10 acts as a suppressor of neutrophil pyroptosis, and its reduced expression exacerbates pyroptosis-driven sepsis progression	[82]
Sepsis-associated encephalopathy	ac4C acetylation	GABABR1	NAT10 promotes GABABR1 expression through mRNA acetylation	NAT10 promotes cognitive dysfunction in sepsis	[83]
Sepsis-induced pulmonary injury	ac4C acetylation	TFRC	NAT10 promotes TFRC expression through mRNA acetylation	NAT10 promotes sepsis-induced pulmonary injury	[84]
Metabolic Dysfunction-Associated Steatotic liver Disease (MASLD)	ac4C acetylation	Srebp-1c	NAT10 stabilizes Srebp-1c mRNA and upregulates lipogenic enzymes	NAT10 exacerbates high-fat-diet-induced liver steatosis	[85]
Metabolic Dysfunction-Associated Steatotic liver Disease (MASLD)	ac4C acetylation	CD36、FATP2	NAT10 stabilizes CD36 and FATP2 mRNA	NAT10 promotes maternal high-fat diet-induced hepatic steatosis	[86]
Sindbis virus replication	ac4C acetylation	LY6E	NAT10 regulates mRNA stability and translation efficiency of LY6E via the 3'-UTR region	NAT10 promotes sindbis virus replication	[62]
enterovirus 71 replication	ac4C acetylation	enterovirus 71 genome	NAT10 enhances viral RNA translation via selective recruitment of PCBP2 and increases the binding of RNA-dependent RNA polymerase to viral RNA	NAT10 promotes virus replication	[87]
Kaposi's sarcoma-associated herpesvirus reactivation	ac4C acetylation	tRNA	NAT10 functions to increase the ac4C level of tRNA^Ser−CGA−1–1^	NAT10 promotes translation efficiency of viral lytic genes, and virion production	[51]
Pulmonary Fibrosis	ac4C acetylation	TGFB1	NAT10 enhances the stability of TGFB1 mRNA	NAT10 promotes pulmonary EMT and fibrosis	[88]
Wound Repair	-	-	NAT10 compromises the level of nuclear p65 by facilitating its poly-ubiquitination, thus accelerates its degradation in the nucleus	NAT10 promotes cutaneous wound repair	[89]
Hutchinson-Gilford Progeria Syndrome (HGPS)	-	-	NAT10 promotes health decline of HGPS mice	NAT10 shortens lifespan and accelerates aging syndrome	[19]
Hutchinson-Gilford Progeria Syndrome (HGPS)	-	-	NAT10 affects nuclear architecture	NAT10 takes part in misshaped nuclear, altered chromatin organization	[20]
Neuropathic Pain	ac4C acetylation	Vegfa	NAT10 enhances the stability of Vegfa mRNA	NAT10 contributes to the central sensitivity and neuropathic pain induced by spared nerve injury	[90]
Eriodontitis	ac4C acetylation	KLF6	NAT10 enhances the stability of KLF6 mRNA	NAT10 promotes periodontal inflammation	[91]
Osteoclastogenesis	ac4C acetylation	Fos	NAT10 enhances the stability of Fos mRNA	NAT10 promotes osteoclastogenesis	[92]

### NAT10 in cancer

NAT10-mediated ac4C modification has emerged as a critical driver of tumorigenesis, with elevated NAT10 expression observed in 92% of cancers and strongly correlating with poor prognosis across malignancies [93–102]. Mechanistically, NAT10 orchestrates a multifaceted oncogenic program through ac4C-dependent stabilization and translation of key oncogenic transcripts. For example, NAT10 enhances EGFR translation efficiency in esophageal cancer by enriching ac4C-modified tRNAs, thereby activating MAPK/ERK signaling [103]. In hepatocellular carcinoma, ac4C modification of HMGB2 mRNA promotes eEF2 binding and translation elongation, driving tumor growth and metastasis [102]. Similarly, NAT10 stabilizes BCL9L, SOX4, and AKT1 transcripts in bladder cancer through 3'UTR ac4C modification, fueling proliferation and invasion [104]. Notably, NAT10 extends its regulatory reach beyond protein-coding RNAs—ac4C modification of lncRNA SIMALR enhances its stability, enabling activation of eEF1 A2-mediated ITGB4/ITGA6 translation in nasopharyngeal carcinoma [68].

NAT10 intersects with other epigenetic layers to amplify oncogenic signaling. In gastric cancer, NAT10-mediated SRSF2 stabilization induces exon skipping in YTHDF1 pre-mRNA, creating a truncated m6 A reader isoform that promotes tumor progression [105]. This epitranscriptomic crosstalk suggests NAT10 coordinates with m6 A machinery to optimize oncogene expression. Furthermore, NAT10’s lysine acetyltransferase activity contributes to chemoresistance in hepatocellular carcinoma, NAT10 acetylates ACLY to boost nuclear acetyl-CoA production, enabling transcriptional activation of drug resistance genes [74]. This dual RNA/protein acetylation capacity positions NAT10 as a master regulator of cancer cell plasticity.

The tumor microenvironment undergoes NAT10-driven remodeling through metabolic and immune modulation. In breast cancer, NAT10-mediated stabilization of JunB mRNA upregulates LDHA expression, creating an immunosuppressive niche via lactate-driven inhibition of cytotoxic T cells [106]. Concurrently, NAT10 acetylates NPM1 to enhance PD-L1 expression, facilitating immune evasion [107].

Clinical evidence underscores NAT10’s translational relevance. NAT10 overexpression predicts reduced 5-year survival in HCC patients [108] and positively correlates with immune checkpoint proteins [107]. Therapeutic targeting of NAT10 with remodelin synergizes with sorafenib to overcome ferroptosis resistance in nasopharyngeal carcinoma by destabilizing SLC7 A11 transcripts [109], while combinatorial inhibition of NAT10 and MDM2 restores wild-type p53 activity in gastric cancer [110]. These findings establish NAT10 as a central node in cancer pathogenesis and therapy resistance.

### NAT10 in autoimmune and inflammatory diseases

**Fig. 2:**
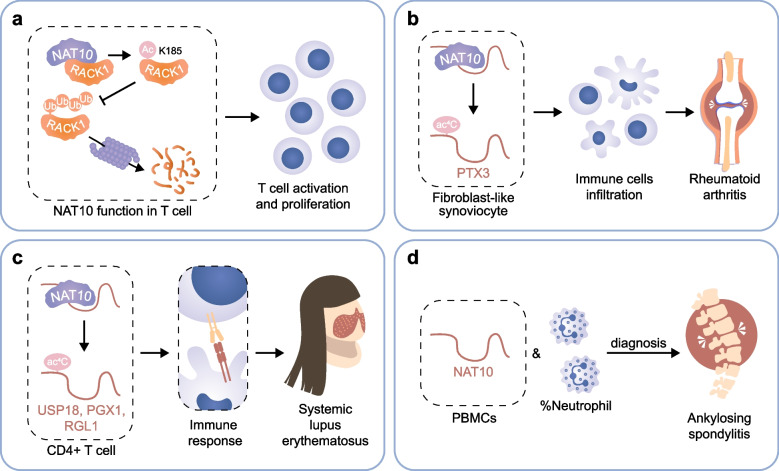
Mechanisms of NAT10 in autoimmune and inflammatory diseases. **a** NAT10 acetylates RACK1 at K185, thereby inhibiting the ubiquitination and proteasome degradation of RACK1 in T cells, leading to T cell activation and proliferation. **b** ac4C acetylation of PTX3 by NAT10 in fibroblast-like synoviocyte promotes immune cell infiltration and progression of Rheumatoid Arthritis. **c** NAT10 mediates ac4C modification of USP18, PGX1 and RGL1 mRNAs in CD4 + T cells and promotes immune response in Systemic Lupus Erythematosus. **d** The diagnosis model of NAT10 mRNA with neutrophil percentage in PBMC in Ankylosing spondylitis

Autoimmune and inflammatory diseases encompass a spectrum of disorders driven by immune homeostasis breakdown, predominantly mediated through dysregulated T-cell responses [111, 112]. Diseases like rheumatoid arthritis (RA), systemic lupus erythematosus (SLE) are examples of such dysregulation. In these cases, abnormal hyperactivity of T-cells and uncontrolled proliferation result in tissue damage and inflammatory responses [113, 114]. Autoreactive T-cells mistakenly identify self-antigens as foreign substances, which triggering an aberrant immune response that targets host tissues and results in progressive tissue damage [115]. In these autoimmune and inflammatory diseases, NAT10 serves as a critical modulator of T cell cycle progression (Fig. 2). NAT10 regulated T cell activation and proliferation upon antigen stimulation by acetylating RACK1 at K185 and preventing subsequent RACK1 K48-linked ubiquitination and degradation, leading to enhanced supply of energy and biosynthetic precursors and, eventually, T cell proliferation [116]. The result highlights the essential function of NAT10 in T cell self-renewal and metabolism and proposes NAT10 for the potential development of novel therapies for immune-related disorders.

NAT10 and ac4C modification have potential application values in targeted therapy and clinical diagnosis of autoimmune and inflammatory diseases. In rheumatoid arthritis (RA), elevated levels of NAT10 and ac4C were found in synovium from RA patients. NAT10 mediated ac4C modification could promote rheumatoid synovial aggression and inflammation, which could be reversed by NAT10 inhibitor [79]. In systemic lupus erythematosus (SLE), mRNA ac4C modification have been pointed out as potential therapeutic targets in SLE pathogenesis. The unique ac4C-related transcripts in CD4^+^ T cells of SLE patients, including USP18, GPX1, and RGL1, regulating mRNA catabolic processes and translational initiation, played essential roles in immune and inflammatory responses of SLE processes [80]. In ankylosing spondylitis, NAT10 mRNA together with neutrophil percentages could be employed to construct a novel predictive model to distinguish new-onset ankylosing spondylitis patients from RA and SLE [81].

### NAT10 in infectious diseases

**Fig. 3:**
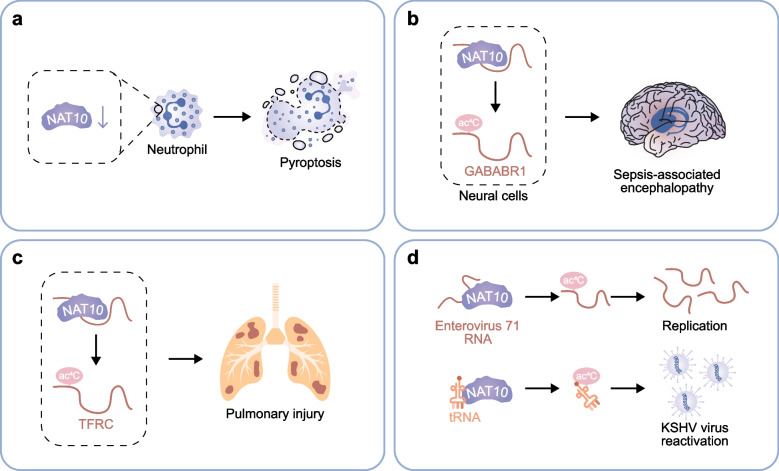
Mechanisms of NAT10 in infectious diseases. **a** In sepsis, the decreased expression of NAT10 in neutrophils induces pyroptosis. **b** ac4C acetylation of GABABR1 by NAT10 in neural cells promotes sepsis-associated encephalopathy. **c** NAT10 mediates ac4C modification of TFRC mRNA which promotes sepsis-associated pulmonary injury. **d** NAT10 mediates ac4C modification of enterovirus 71 RNA and tRNA for KSHV virus, promoting virus replication and reactivation

NAT10 and ac4C modifications are deemed to have crucial roles in infectious diseases [117, 118] (Fig. 3). The mechanism underlying the regulation of NAT10 in infectious diseases was reported in sepsis and in virus replication and reactivation. Sepsis is a life-threatening organ dysfunction which is brought about by an uncontrolled host responses that are induced by microbial invasion. The impaired functionality of neutrophils at infection sites, combined with aberrant immune activation, has been extensively documented as a critical contributor to compromised pathogen clearance in infectious diseases [119–123]. Neutrophils exhibit a dual role, mediating protective inflammatory responses to pathogens while acting as key instigators of tissue injury in septic conditions [124, 125]. Neutrophil-specific NAT10 expression was found the negative regulator of neutrophil pyroptosis. The decreased expression of NAT10 in neutrophils contributes to the progress of sepsis by promoting neutrophil pyroptosis [82]. Research indicates that the neutrophil-specific NAT10 contributes to the progress of sepsis, and revealing a potential therapeutic target of downstream pathways for sepsis. Sepsis-associated encephalopathy (SAE) and sepsis-associated acute lung injury are both acute organ dysfunctions induced by sepsis. SAE stands as a major neurological manifestation of sepsis, significantly driving elevated mortality rates and deteriorating clinical outcomes in affected patients. In cecal ligation and puncture-induced SAE, NAT10 promotes GABABR1 expression through mRNA acetylation, leading to cognitive dysfunction. Neuronal-specific NAT10 knockdown improved cognitive function, highlighting its critical role in SAE [83]. Acute lung injury is one of the most common complications of sepsis, which is difficult to treat and has a high fatality rate [126]. NAT10 was also found promoting sepsis-induced pulmonary injury by mediating ac4C acetylation of TFRC mRNA [84]. NAT10 inhibition could alleviate sepsis-induced pulmonary injury. In this way, NAT10 was involved in sepsis related complications and contributing to the occurrence and progression of complications.

Ac4C modification in the regulation of virus infection has also been investigated. NAT10 could regulate virus functions through ac4c modification in the host mRNAs and viral genome. In the previous mechanism, alphavirus infection induced cellular NAT10 upregulated, and ac4C modifications were promoted in turn to enhance alphavirus replication [62]. In the later mechanism, 5'UTR of the enterovirus 71 genome was ac4C modified by the host acetyltransferase NAT10. Ac4C modification promoted viral RNA translation through the selective recruitment of PCBP2 to the IRES and increased RNA stability. Ac4C-deficient enterovirus 71 virus reduced pathological damage in mice [87]. NAT10 also exerts its RNA acetyltransferase function and increases the ac4C modification of tRNA to eventually promote the viral yield and latency-to-lytic cycle transition of KSHV, a cancer-promoting herpesvirus [51]. The ac4C modification of host mRNA and viral RNA identified in these discoveries, which indicates the inhibition of ac4C could be a potential target when developing virus antivirals.

### NAT10 in cardiac diseases

**Fig. 4:**
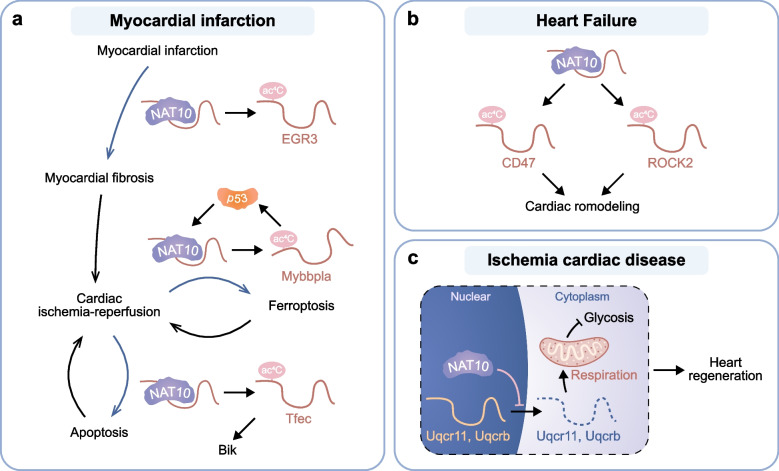
Mechanisms of NAT10 in cardiac diseases. **a** In myocardial infarction, NAT10 mediates ac4C modification of EGR3 mRNA and promotes myocardial fibrosis. In cardiac ischemia–reperfusion injury, p53 activates NAT10-mediated ac4C modification of Mybbpla mRNA and in turn activate p53 itself, which promotes the cellular ferroptosis and progression of cardiac ischemia–reperfusion injury. NAT10 also enhances ac4C modification of Tfec mRNA, which induces the pro-apoptotic protein Bik and further promotes cardiomyocyte apoptosis. **b** In heart failure, the ac4c modification of CD47 and ROCK2 mRNA mediated by NAT10 promotes cardiac remodeling. **c** In ischemia cardiac disease, NAT10 acts independent of acetyltransferase and inhibits the translation of Uqcr11 and Uqcrb by inhibiting their mRNA nuclear export, which blocking mitochondria respiration and upregulating glycolysis

Cardiac disease, also known as cardiovascular disease, is the prevalent cause of mortality around the world [127]. NAT10 contributes to the pathological progression of cardiovascular diseases, including myocardial infarction and heart failure (Fig. 4).

In myocardial infarction, NAT10 may take part in promoting the cardiomyocyte death and myocardial fibrosis to exacerbate cardiac injury. Qu et al. revealed that P53 functions as an endogenous inducer of NAT10 through transcription-dependent mechanisms during cardiac ischemia–reperfusion, thereby driving cardiomyocyte ferroptosis and aggravating ischemia–reperfusion injury [75]. Wang et al. reported that NAT10 interacts with a heart-apoptosis-associated piRNA and enhances ac4C acetylation of Tfec mRNA transcript, which further promote the progression of cardiomyocyte apoptosis [76]. Hao et al. reported that NAT10 is highly expressed in infarcted myocardial tissue, inhibition of NAT10 can alleviate myocardial fibrosis after myocardial infarction [77]. These studies demonstrate targeting NAT10 may be a novel approach for treating ischemia heart diseases. In the final stages of cardiac disease development, the heart is unable to pump blood efficiently to meet the body's metabolic needs, leading to a buildup of blood in the heart and other parts of the body and causing heart failure. NAT10 is involved in the process of heart failure especially by promoting cardiac remodeling. Cardiac remodeling, a hallmark of heart failure, is mediated by NAT10-mediated ac4C acetylation modification. NAT10 could aggravate the process of heart remodeling by upregulating mRNA ac4C modification of CD47 and ROCK2 and enhancing the stability and translation. The administration of remodelin has been shown to prevent cardiac functional impairments by suppressing cardiac fibrosis, hypertrophy, and inflammatory responses. NAT10 may be a promising therapeutic target against cardiac remodeling and heart failure. Emerging evidence indicates that genetic depletion of NAT10 triggers cardiomyocyte apoptosis, a critical determinant in the progression of heart failure pathophysiology [128]. It is worth noting that, NAT10 was also found playing a non-classic role in cardiac disease independent of the ac4C catalyzing activity [78]. NAT10 in cardiomyocytes binds to Uqcr11 and Uqcrb respective transcripts, inhibits their mRNA nuclear export, and reduces the translation of their mRNA in ribosomes, promoting mouse cardiac regeneration and improves cardiac function after injury. Inhibiting NAT10 with remodelin or genetically removing NAT10 from cardiomyocytes both inhibit heart regeneration.

### NAT10 in metabolic liver disease

**Fig. 5:**
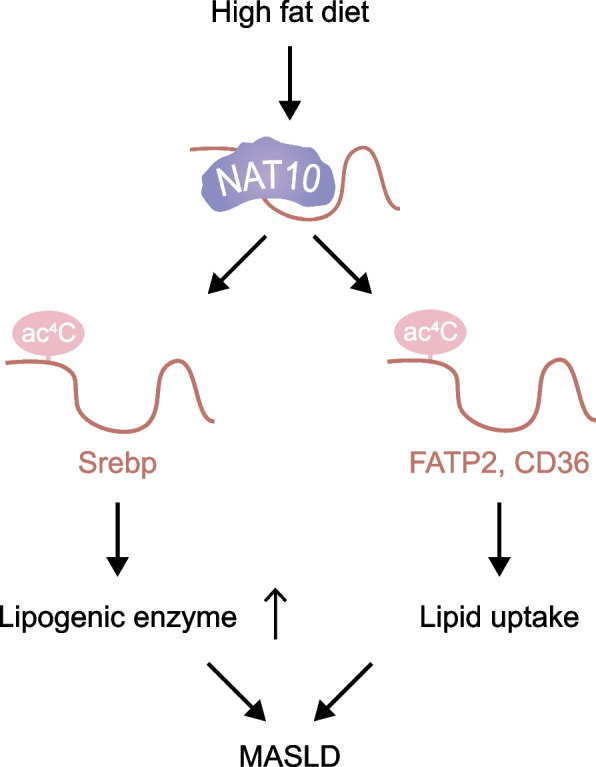
Mechanisms of NAT10 in metabolic liver disease. NAT10 regulates lipid metabolism in MASLD by enhancing the mRNA stability of CD36, FATP2 and Srebp-1c

Metabolic dysfunction-associated steatotic liver disease (MASLD) is quickly becoming a highly common chronic liver disorder, which is estimated to be 25% worldwide. Research has discovered that NAT10 could exacerbate high-fat-diet-induced liver steatosis, whereas knockout of NAT10 could protect from diet-induced hepatic steatosis and steatohepatitis (Fig. 5). Mechanically, NAT10 could regulate lipid metabolism in MASLD by stabilizing Srebp-1c mRNA and upregulating lipogenic enzymes [85]. NAT10 could also regulate lipid uptake by enhancing the mRNA stability of CD36 and FATP2 [86]. These results highlight NAT10 as a potential therapeutic targets for MASLD.

### NAT10 in other diseases

In addition to its well-documented functions, NAT10 plays a significant role in a diverse array of diseases through its enzymatic activity in ac4C modification. In pulmonary fibrosis, NAT10 accelerates disease progression by stabilizing transforming growth factor beta 1 (TGFB1) mRNA in an ac4C-dependent manner, promoting epithelial-to-mesenchymal transition (EMT) and fibrosis upon exposure to ambient particulate matter [111]. Conversely, NAT10 exhibits a protective role in wound repair, where it enhances keratinocyte migration and cutaneous healing via the NF-κB-IL-6 axis, facilitating nuclear retention of p65 and subsequent IL-6/IL-8-STAT3 signaling activation [112].

In Hutchinson-Gilford Progeria Syndrome (HGPS), a premature aging disorder, NAT10 inhibition with the small molecule remodelin ameliorates nuclear defects and extends healthspan in mouse models by restoring chromatin organization and reducing DNA damage [10, 11]. Similarly, NAT10 contributes to neuropathic pain by catalyzing ac4C modification of Vegfa mRNA, enhancing its translation efficiency and driving central sensitization in spinal neurons [113].

NAT10 also exacerbates inflammatory bone loss through ac4C-mediated stabilization of Fos mRNA, which activates MAPK signaling and promotes osteoclastogenesis. Pharmacological inhibition of NAT10 reduces bone resorption in periodontitis and inflammatory osteolysis models [114]. Furthermore, in periodontal inflammation, Ropinirole, a dopamine receptor agonist, suppresses LPS-induced damage by inhibiting NAT10 activity, thereby reducing KLF6 mRNA stability and inflammatory cytokine production [115].

## NAT10 in cancer biology: mechanisms and therapeutic implications

While NAT10 dysregulation exerts broad pathological effects across diverse diseases, its role in cancer biology represents a unique convergence of multifaceted oncogenic mechanisms. Unlike its disease-specific functions in single pathological processes (e.g., neutrophil pyroptosis regulation in sepsis or lipid metabolism modulation in MASLD), NAT10 in cancer operates as a central signaling hub that simultaneously coordinates transcriptional, post-transcriptional, and post-translational modifications. The following section dissects how NAT10 acts as both a master regulator of malignant progression and a promising pan-cancer therapeutic target.

**Fig. 6:**
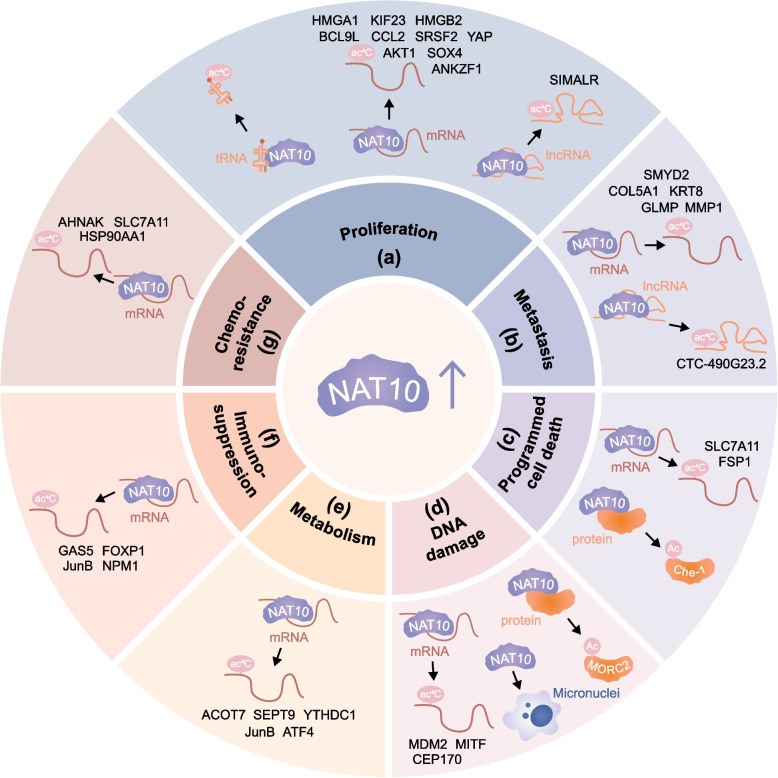
The function of NAT10 in cancer progression. **a** NAT10 promotes cancer proliferation by ac4C modification of mRNAs of HMGA1, KIF23, HMGB2, BCL9L, CCL2, SRSF2, AKT1, SOX4, ANKZF1, YAP, by ac4C modification of tRNA which enhances EGFR mRNA translation, and by ac4C modification of lncRNA SIMALR. **b** NAT10 promotes cancer metastasis by ac4C modification of mRNAs of COL5 A1, KRT8, SMYD2, GLMP, MMP1, and by ac4C modification of lncRNA CTC-490G23.2. **c** NAT10 regulates several programmed cell death pathways by ac4C modification of mRNAs of FSP1 and SLC7 A11 and by acetylation of Che-1 protein at K228. **d** NAT10 regulates DNA damage by ac4C modification of mRNAs of MDM2, MITF, CEP170, by lysine acetylation of MORC2, and by inducing micronuclei formation. **e** NAT10 regulates metabolism by ac4C modification of mRNAs of ACOT7, SEPT9, YTHDC1, JunB and ATF4. **f** NAT10 promotes the immunosuppressive tumor microenvironment formation by ac4C modification of mRNAs of GAS5, FOXP1, JunB and NPM1. **g** NAT10 promotes chemoresistance by ac4C modification of mRNAs of HSP90 AA1, AHNAK, SLC7 A11

Specifically, the aberrant activation of NAT10-mediated ac4C have been associated with the progression of tumor malignancies mainly through the regulation of cell proliferation, metastasis, programmed cell death, DNA damage, metabolism, immunosuppression, and chemoresistance. Studies describing the function of NAT10 in cancer progression by acetylating a variety of substrates have been listed (Table 2, Fig. 6).

**Table 2:** Substrates of NAT10 in human cancers

Cancer type	Role	Substrates	Molecular mechanism	Cellular function	Ref
Gastric cancer	ac4C acetylation	MDM2	Stabilizes MDM2 mRNA, leads to MDM2 upregulation and p53 degradation	Promotes cell proliferation and tumor growth, impairs DNA repair, cell cycle arrest and apoptosis	[110]
	ac4C acetylation	COL5 A1	Maintains COL5 A1 mRNA stability and increases its expression	Promotes EMT and metastasis	[95]
	ac4C acetylation	SEPT9	Stabilizes SEPT9 mRNA, activates the HIF-1 pathway	Promotes glucose metabolism reprogramming and glycolysis addiction	[46]
	ac4C acetylation	SRSF2	Stabilization of SRSF2, which directly associates with the pre-mRNA of the m⁶A reader YTHDF1 and leads to an elevation in a shorter variant of the YTHDF1 transcript	Promotes proliferation and migratory capabilities of tumors	[105]
	ac4C acetylation	SMYD2	Maintains SMYD2 mRNA stability	Enhancing the migration and invasion abilities of tumor cells	[129]
Lung cancer	ac4C acetylation	GAS5	Maintains GAS5 mRNA stability and activates type I interferon signaling	Promoting immune cell infiltration	[130]
Nasopharyngeal carcinoma	ac4C acetylation	lncRNA SIMALR	Stabilizes lncRNA SIMALR, which mediates the activation of eEF1 A2 phosphorylation to accelerate the translation of ITGB4/ITGA6	Promotes the proliferation and metastasis of tumors	[68]
	ac4C acetylation	SLC7 A11	Maintains SLC7 A11 mRNA stability, inhibiting sorafenib-induced ferroptosis	Promotes ferroptosis and the growth of sorafenib-resistant tumors	[109]
Colorectal cancer	ac4C acetylation	KIF23	Maintains KIF23 mRNA stability, activates Wnt/β-catenin pathway	Inhibits apoptosis and enhances cell proliferation, migration, and invasion	[131]
	ac4C acetylation	FSP1	Stabilizes FSP1 mRNA	Inhibits ferroptosis, promotes cell proliferation and metastasis	[94]
	unknown	unknown	Increases micronuclei formation and senescence-associated secretory phenotype pathway activation	Promotes cell stress response and carcinogenesis	[24]
Ovarian cancer	ac4C acetylation	ACOT7	Maintains ACOT7 mRNA stability, modulates fatty acid metabolism	Promotes tumor progression by suppressing ferroptosis	[100]
Esophageal cancer	ac4C acetylation	tRNA	Enhances the translation efficiency of EGFR	Promotes gefitinib resistance	[103]
	ac4C acetylation	CTC-490G23.2	Enhances CTC-490G23.2 stabilization and expression	Promotes cancer invasion and metastasis	[69]
Bladder cancer	ac4C acetylation	BCL9L, SOX4, AKT1	Enhances the mRNA’s stability and protein expression	Promotes cell proliferation, migration, invasion, survival	[104]
	ac4C acetylation	AHNAK	Stabilizes AHNAK mRNA by protecting it from exonucleases	Promotes cisplatin chemoresistance	[47]
Multiple myeloma	ac4C acetylation	CEP170	Enhances CEP170 translation efficiency	Promotes cell proliferation and chromosomal instability	[132]
Hepatocellular carcinoma	ac4C acetylation	HSP90 AA1	Maintains HSP90 AA1 mRNA stability and upregulates its expression	Enhances metastasis ability and apoptosis resistance	[133]
	ac4C acetylation	HMGB2	Enhances HMGB2 translation efficiency	Promotes cell growth and metastasis	[102]
	ac4C acetylation	MDM2	Maintains MDM2 mRNA stability, which regulates mutant p53 ubiquitination and stability	Enhance the tumorigenesis	[108]
	Lysine acetylation	ACLY	Acetylates ACLY and activates the transcription of CYP2 C9 and PIK3R1	Promotes chemoresistance	[74]
Melanoma	Unknown	MITF	Unknown	Promotes melanogenesis and melanoma growth	[134]
Breast cancer	Lysine acetylation	MORC2	Stabilizes MORC2 mRNA transcripts and response to DNA damage	Promotes resistance to DNA damage drugs	[55]
	ac4C acetylation	JunB	Promotes JunB mRNA stability, which further up-regulates LDHA expression	Increases glycolysis and creates an immunosuppressive tumor microenvironment	[106]
	Lysine acetylation	NPM1	Promotes acetylation of NPM1 and subsequent increase in PD-L1 expression	Promotes immunosuppressive tumor microenvironment	[107]
	ac4C acetylation	ELOVL6, ACSL1, ACSL3, ACSL4, ACDSB, ACAT1	Stabilizes ELOVL6, ACSL1, ACSL3, ACSL4, ACDSB, ACAT1 mRNA transcripts	Promotes lipid accumulation and influences tumour invasion and metastasis	[93]
Cervical cancer	ac4C acetylation	FOXP1	Enhances FOXP1 mRNA stability and its translation efficiency	Promotes malignant progression and facilitates immunosuppression	[135]
	ac4C acetylation	YAP	Enhances YAP mRNA stability	Promotes cell growth and metastasis	[136]
Oral cancer	ac4C acetylation	MMP1	Enhances MMP1 mRNA stability	Promotes tumorigenesis and metastasis	[101]
Prostate Cancer	ac4C acetylation	HMGA1	Enhances HMGA1 mRNA stability	Promotes cell cycle progression and tumorigenesis	[137]
	ac4C acetylation	KRT8	Enhances KRT8 mRNA stability	Promotes EMT and metastasis	[137]
Osteosarcoma	ac4C acetylation	YTHDC1	Enhances YTHDC1 mRNA stability, which suppress glycolysis pathway in an m^6^A methylation-dependent manner	Promotes growth and motility of tumor cells	[97]

### Proliferation

NAT10-mediated ac4C modification is associated with cancer progression, and inhibiting it can block cancer progression. By employing acetylated RNA immunoprecipitation sequencing (acRIP-seq) and ribosome profiling sequencing (Ribo-seq) approaches, multiple downstream key genes like EGFR [103], BCL9L, SOX4, AKT1 [104], KIF23 [131], HMGA1 [137], HMGB2 [102], SRSF2 [105], ANKZF1 [96], and CCL2 [138] are validated the functional targets of NAT10. Through mediating the ac4C modification of tRNA or mRNA of these genes, NAT10 enhance the translation efficiency [103, 104] and promote cancer progression. In esophageal cancer, NAT10 enhances the abundance of ac4 C-modified tRNAs and further enhances the translation efficiencies of EGFR [103], which is a member of receptor tyrosine kinase family and is activated by various ligands to mediate various cellular activities to promote cancer progression. In colorectal cancer, NAT10 mediates the stability of KIF23 mRNA by binding to its mRNA 3'UTR region and up-regulating its mRNA ac4c modification. The upregulation of KIF23 activates Wnt/β-catenin pathway and more β-catenin is transported into the nucleus which led to colorectal cancer progression [131]. high mobility group AT-hook 1 (HMGA1) and high mobility group protein B2 (HMGB2) have the ability to advance several key pathways, like Wnt/β-catenin pathway, the phosphatidylinositol 3 kinase/protein kinase B (PI3 K/Akt) cascade, the Hippo signaling pathway, and the MAP kinase-ERK kinase/extracellular-signal-regulated kinase (MEK/ERK) pathway, all of which raise the malignancy of tumor cells [139–141]. HMGB2 demonstrates oncogenic activity across multiple cancer types, with its pronounced overexpression in HCC directly accelerating malignant transformation [141–143]. NAT10 enhances the stability of HMGA1 by acetylating its mRNA, thereby promoting cell cycle progression to improve cell proliferation in prostate cancer [137]. NAT10-mediated ac4C modification of HMGB2 mRNA, and the ac4C sites in the CDS of HMGB2 mRNA enhance eEF2 binding, promoting translation elongation of HMGB2 mRNA, thereby critically driving HCC progression and metastatic dissemination [102]. In gastric cancer, NAT10 engages with the splicing factor serine/arginine-rich splicing factor 2 (SRSF2), instigating its acetylation and subsequent stabilization. The acetylated form of SRSF2 directly associates with the pre-mRNA of the m⁶A reader YTHDF1, prompting an increase in the skipping of exon 4 within YTHDF1’s pre-mRNA. This leads to an elevation in a shorter variant of the YTHDF1 transcript, which has the capacity to invigorate the proliferation and migratory capabilities of gastric cancer cells [105]. Studies revealed ANKZF1, a zinc finger tRNA hydrolase, as an additional regulatory target in NAT10's functional network. The ac4C-driven upregulation of ANKZF1 enhances its competitive binding to YWHAE (14–3-3ε), thereby disrupting YWHAE-mediated cytoplasmic sequestration of YAP1 and subsequently promoting the malignant progression of clear-cell renal cell carcinoma [96]. By binding to CCL2 messenger RNA, NAT10 escalates the expression level of the CCL2 protein within intrahepatic cholangiocarcinoma and in their extracellular matrix. This promotes the proliferation of intrahepatic cholangiocarcinoma cells and steers the polarization of macrophages towards the M2 phenotype [138].

In addition to the regulation of downstream protein mRNA levels, NAT10 also promotes the ac4C modification of long non-coding RNAs. The stability of lncRNA SIMALR is enhanced by NAT10-mediated acetylation and its overexpression in nasopharyngeal carcinoma functions as the oncogenic role [68].

Beyond the downstream target proteins of NAT10 reported, there are also upstream regulatory proteins and interacting proteins regulating NAT10 function found. RNA-binding protein with serine-rich domain 1 (RNPS1) is a protein known for its role in the post-transcriptional modification of mRNA, which includes pre-mRNA splicing, mRNA export from the nucleus to the cytoplasm, and the stabilization of mRNA molecules [144]. RNPS1 directly interacts with NAT10, inhibiting the ubiquitination degradation of NAT10 by E3 ubiquitin ligase, zinc finger SWIM domain-containing protein 6 (ZSWIM6), thereby promoting translation process of genes regulating malignant progression in head and neck squamous cell carcinoma [145]. Circular RNA circMAST1 competitively binds to NAT10 and obstructing NAT10-mediated ac4C modification on Yes-associated protein (YAP) mRNA, which accelerates YAP mRNA degradation and subsequently curtails tumor advancement in cholangiocarcinoma [136]. In summary, NAT10 promotes tumor proliferation by mediating ac4C modification of key oncogenes, enhancing mRNA stability and translation efficiency to drive cancer progression.

### Metastasis

The regulatory role of NAT10 in tumor metastasis and cell motility was reported in gastric cancer, head and neck squamous cell carcinoma, prostate cancer, oral squamous cell carcinoma and esophageal squamous cell carcinoma. By inducing the ac4C modification and stability of epithelial-mesenchymal transition (EMT) markers, NAT10 plays a crucial role in promoting cancer metastasis. NAT10 is reported to promote gastric cancer metastasis by regulating ac4C acetylation on 3’UTR mRNA of COL5 A1, which is a marker of EMT [146, 147], to maintain its mRNA stability and further promotes EMT indirectly [95]. NAT10 also mediates the ac4C modification of lncRNA CTC-490G23.2, which leads to the stabilization and overexpression of CTC-490G23.2 in primary esophageal squamous cell carcinoma and its further upregulation in metastatic tissues. CTC-490G23.2 serves as a critical scaffold, enhancing the interaction between CD44 pre-mRNA and polypyrimidine tract-binding protein 1 (PTBP1), which triggering an oncogenic shift in splicing from the conventional isoform to the variant capable of binding to EMT marker vimentin [69]. Keratin 8 (KRT8), predominantly localized to the basal domain of epithelial cells, constitutes an essential element of the intermediate filament network critical for cytoskeletal integrity. Notably, KRT8 contributes to the progression of cancer by aiding the EMT process [148]. Specifically, KRT8 acts as a downstream target of NAT10, enabling it to perform mRNA acetylation modifications, which in turn influence the metastasis of prostate cancer [137].

Neutrophil extracellular traps (NETs) are net-like structures composed of DNA-histone complexes and proteins released by activated neutrophils [149, 150]. NETs could increase the mRNA and protein expression levels of NAT10, thus promoting the ac4C modification of SET and MYND domain containing 2 (SMYD2) and its stability, and enhancing the migration and invasion abilities of gastric cells [129]. In head and neck squamous cell carcinoma, NAT10 promotes lymph node metastasis and remodels the malignant niche surrounding head and neck squamous cell carcinoma cells via ac4C-dependent stabilization of the glycosylated lysosomal membrane protein (GLMP) transcript, which triggered the activation of the MAPK/ERK signaling pathway [99]. In oral squamous cell carcinoma, lentivirus-mediated NAT10 knockdown markedly suppressed cell migration and invasion. MMP1 was validated a potential target of NAT10 [101], which is responsible for degradation of the basement membrane and the connective tissue for distant metastasis of epithelial tumors [151]. Overall, NAT10 catalyzes ac4C deposition on its target mRNAs, enhancing their stability and translation efficiency to regulate downstream protein synthesis, leading to EMT transition and tumor metastasis. These pivotal molecules present promising targets for inhibiting tumor metastasis.

### Programmed cell death regulation

Programmed cell death can be classified into six major types: ferroptosis, autophagy, pyroptosis, apoptosis, necroptosis and cuproptosis. NAT10 has been reported to regulate several programmed cell death pathways by acetylation of key proteins in them. By promoting the acetylation and stability of the proteins, NAT10 acts as a cell death inhibitor and cell survival promoter. Ferroptosis is a unique type of regulated cell death characterized by the iron-dependent accumulation of lipid peroxides. Ferroptosis is caused by a redox imbalance between the production of oxidants and antioxidants, which is driven by the abnormal expression and activity of multiple redox-active enzymes that produce or detoxify free radicals and lipid oxidation products [152]. The ferroptosis-induced antioxidant activity depends on three systems, which are as follows: DHODH/ubiquinol, FSP1/NAD(P)H/ubiquinol, and SLC7 A11/GSH/PLOOH [153, 154]. Studies have demonstrated that NAT10 restrains ferroptosis by stabilizing the mRNA of the ferroptosis suppressor protein 1 (FSP1) [155] and solute carrier family 7 member 11 (SLC7 A11) via ac4C modification, preventing oxidative stress that triggers phospholipid oxidation to initiate ferroptosis, thereby promoting the progression of cancer [94, 109].

NAT10 has also been reported to regulate autophagy and pyroptosis, although not in cancers. Autophagy functions as a lysosome-dependent catabolic pathway through which cellular components such as proteins and organelles are selectively degraded and recycled to preserve homeostatic balance. Dysregulation of autophagic activity often culminates in programmed cell death [156, 157]. NAT10 could regulate the metabolic switch between rRNA biogenesis and autophagy, which depends on the role of lysine acetyltransferase. Acetylation of NAT10 could activate rRNA biogenesis, whereas deacetylation of NAT10 by SIRT1 shifts the cellular balance from rRNA biogenesis to autophagy. Under adequate energy supply conditions, NAT10 is acetylated to activate rRNA biogenesis, meanwhile binding to and acetylates Che-1 at K228 to suppress the Che-1-mediated autophagy induction. Upon energy stress, NAT10 is deacetylated by Sirt1, leading to suppression of NAT10-activated rRNA biogenesis [52]. Targeting NAT10's acetylation-dependent regulation of these pathways represents a potential strategy to overcome therapy resistance and enhance tumor cell death.

### DNA damage response

NAT10 was demonstrated to inhibit cell cycle arrest under DNA damage condition. p53, a famous tumor suppressor gene, maintains genome stability by arresting cells for damage repair or triggering programmed cell death to eradicate compromised cells when responding to genotoxic stresses. As an acetyltransferase, NAT10 could both regulate the wildtype p53 and mutant p53 expressions to promote cancer progression. NAT10 catalyzes ac4C acetylation on MDM2 mRNA, stabilizing this transcript and upregulating MDM2 protein expression. As MDM2 is a known ubiquitin ligase for wildtype p53, its increased levels lead to the ubiquitination and subsequent degradation of wildtype p53, effectively downregulating wildtype p53 in gastric cancer [110]. NAT10 also stabilizes the expression of mutant p53 by counteracting the Mdm2-induced ubiquitination and degradation of mutant p53 in hepatocellular carcinoma [108]. This downregulation of wildtype p53 and the stabilization of mutant p53 both contribute to the facilitation of carcinogenesis by allowing for uncontrolled cell proliferation and evasion of apoptosis. The transcription factor MITF plays a non-transcriptional role in suppressing the DNA damage response in melanoma cells [158]. NAT10-silenced or NAT10 inhibitor remodelin-treated cell lines show downregulated MITF expression along with p21 decreases. NAT10 promotes malignant melanoma growth through MITF/cyclinD1/CDK2/p21-mediated cell cycle progression [134]. CEP170, a centrosome protein involved in microtubule organization and assembly, whose abnormalities can lead to mitotic spindle defects causing aneuploidy and chromosomal instability. NAT10 improved the translation efficiency of CEP170 by acetylating CEP170 mRNA, which regulating CEP170 during cell mitosis to influence in multiple myeloma progression [132].

Micronuclei (MN) formation is a pivotal sign of DNA damage and genetic instability [159, 160]. Under oxidative stress condition, NAT10 could be stimulated and promote MN formation by accelerating DNA replication and DNA damage in colorectal cancer [24], indicating that NAT10 not only participates in DNA damage repair but also potentially contributes to its initiation phase.

### Metabolism reprogramming

Regarding the role of NAT10 in metabolism, the current findings point to the regulation of glucose metabolism, fatty acid metabolism and amino acid biosynthesis by NAT10. Through high-throughput sequencing analysis of NAT10-depleted cancer cells, fatty acid metabolism emerged as the top enriched pathway among differential downregulation genes [93], suggesting NAT10 plays a positive regulatory role in fatty acid metabolism. NAT10 facilitates fatty acid metabolism by mediating the ac4C mRNA acetylation and subsequent stabilization of proteins involved in fatty acid metabolism, including ELOVL6, ACSL1, ACSL3, ACSL4, ACADSB, ACAT1 and ACOT7 [100]. In NAT10-depleted cancer cells, the downregulation of overall lipid content, triglycerides and total cholesterol were detected [93]. The same regulatory effect of NAT10 also occurs in glucose metabolism. NAT10 facilitates glycolysis by increasing ac4C modification levels of glucose metabolism reprogramming genes LDHA, SEPT9 [46], YTHDC1 [97] and JunB [106] mRNA. SEPT9, YTHDC1, and JunB have been identified as potential ac4C target genes involved in regulating the metabolic shift. SEPT9 belongs to a highly conserved GTP‐binding cytoskeleton protein family. NAT10 was reported to regulate glucose metabolism reprogramming through regulating SEPT9, which in turn regulates HIF-1 pathway activation in gastric cancer [46]. NAT10 was also reported to regulate lactate production and pyruvate content through YTHDC1, which is ac4C modified by NAT10 and mediated m6 A methylation of PFKM and LDHA RNAs in osteosarcoma [97]. JunB is a crucial member of dimeric activator protein-1 (AP-1) complex, which has been reported to have regulatory functions in glycolytic metabolism [161]. NAT10 upregulated JunB expression by increasing ac4C modification levels on its mRNA, further up-regulated LDHA expression and facilitated glycolysis in breast cancer [106].

Enzyme asparagine synthetase catalyzes the transfer of the amino group from glutamine to the carboxyl group of aspartate, resulting in the formation of asparagine and glutamate. Asparagine metabolism is associated with cancers, where it can contribute to tumor growth and survival. In osteosarcoma, NAT10 enhances mRNA stability of activating transcription factor 4 (ATF4) through ac4C modification [162]. ATF4 induces the transcription of asparagine synthetase, which catalyzes asparagine biosynthesis, facilitating osteosarcoma progression. These results suggests that NAT10 can be a therapeutic target in metabolism.

### Immune evasion

The initiation, growth, and metastasis of cancers are greatly modulated by tumor microenvironment, which comprises neoplastic cells, infiltrating immunocytes, cancer-associated fibroblasts and the extracellular matrix, all of which collectively orchestrate the tumor's behavior and response to therapeutic interventions [163]. Understanding the role of NAT10 in the tumor microenvironment will help facilitate the development of targeted drugs. Researches have unveiled that NAT10 exerts immunosuppressive effects within the tumor microenvironment, primarily via three mechanisms. The first mechanism is the recruitment effect of NAT10 on immune cells, promoting the infiltration of immune cells in the tumor microenvironment. The second mechanism involves the activation of the glycolysis signaling pathway, which subsequently inhibits immune function, the last mechanism entails upregulating the immune checkpoint protein PD-1, thereby suppressing immune responses. In the first mechanism, NAT10 mediates ac4C modification of GAS5, activating type I interferon signaling, which increases the production of CXCL10 and CCL5, further promoting immune cell infiltration. This regulatory mechanism indicates that GAS5 or NAT10 is a promising predictive marker and potential therapeutic target for combination therapy in non-small cell lung cancer [130]. In the second mechanism, tumors display elevated glycolytic activity and upregulated release of lactate will generate an acidic immunological microenvironment. This acidic microenvironment suppresses the cytotoxicity and effector capabilities of immune cells, effectively hindering their antitumor functions [164, 165]. NAT10 induces glycolysis and immunosuppressive tumor microenvironment by upregulating the upstream genes of glycolysis such as FOXP1 and JunB by stimulating ac4C modification of their mRNAs [106, 135]. The stability of transcription factor FOXP1 results in GLUT4 and KHK expression. Notably, GLUT4 serves as a key glucose transporter, facilitating the acceleration of glucose uptake, while KHK kinase is a member of ribokinase superfamily responsible for converting fructose into fructose-1-phosphate in the rate‐limiting first step of fructose metabolism. NAT10 also upregulates JunB expression by increasing ac4C modification levels on its mRNA, which further upregulates LDHA expression and facilitated glycolysis and the immunosuppressive properties of tumor-infiltrating regulatory T cells. In the last mechanism, NAT10 was identified as a facilitator of nucleophosmin (NPM1) acetylation, which enhances the transcription of PD-L1 and inhibits T-cell function in various types of cancer [107]. NAT10 significantly contributes to an immunosuppressive tumor microenvironment and promoting tumor growth, by orchestrating critical pathways including immune cell recruitment, glycolysis enhancement, and PD-1 upregulation, thereby highlighting its pivotal role as a therapeutic target in cancer immunology.

### Therapeutic resistance

NAT10 has been shown to play a significant role in drug resistance across multiple types of cancers. NAT10 upregulates the modification level of HSP90 AA1 mRNA ac4C, upregulating the expression of HSP90 AA1, which further promoting the metastasis of endoplasmic reticulum stress hepatoma cells and the resistance to apoptosis of lenvatinib [133]. These studies identify NAT10 as a novel chemo-resistant driver and the blockage of NAT10 possesses the potential to attenuate HCC chemo-resistance [74]. In bladder cancer, the ac4C modification is induced upon cisplatin treatment and correlates with chemoresistance. NAT10 promotes cisplatin chemoresistance by enhancing DNA damage repair. It binds and stabilizes AHNAK mRNA, and AHNAK -mediated DNA damage repair is required for NAT10-induced cisplatin resistance. Clinically, NAT10 overexpression is associated with chemoresistance, recurrence, and worse clinical outcome [47]. In nasopharyngeal carcinoma, NAT10 expression is upregulated. NAT10 promotes solute carrier family 7 member 11 (SLC7 A11) expression through ac4C acetylation, which inhibits sorafenib-induced ferroptosis in nasopharyngeal carcinoma cells. Sorafenib synergizes with the NAT10 inhibitor remodelin to suppress SLC7 A11 expression and promote ferroptosis [109]. In summary, NAT10 is a key factor in promoting drug resistance in various cancers by affecting target protein stability, and being involved in cell-cycle regulation and DNA repair processes. NAT10 blockade emerges as a potential mechanism-driven strategy to reverse chemoresistance in these tumors.

## Therapeutic potential of NAT10 inhibition

The multifunctional acetyltransferase activity of NAT10 enables diverse therapeutic targeting strategies. By modulating NAT10 activity or expression, researchers have achieved breakthroughs in cancer, fibrosis, neurodegeneration, and anti-aging interventions. This section dissects its therapeutic potential across distinct diseases, supported by mechanistic insights and preclinical evidence.

### Small inhibitors

Remodelin, a small-molecule inhibitor identified via high-throughput screening, targets NAT10's acetyltransferase activity, disrupting its roles in microtubule stabilization and RNA modification. Studies demonstrate that remodelin rescues nuclear morphology defects caused by laminopathies [19, 20], thereby suppressing cancer cell migration and invasion. Remodelin has been widely used in multiple cancers, which includes melanoma [134], multiple myeloma [132], nasopharyngeal carcinoma [109], gastric cancer [46, 110], cervical cancer [135], breast cancer [106], esophageal squamous cell carcinoma [166], osteosarcoma [162] and et al. The combination of NAT10 inhibitors with other therapies—such as sorafenib [109], MDM2 inhibitors [110], CTLA-4 antibody [106, 107], and PD-1/PD-L1 antibody [49] —has shown synergistic antitumor effects (Table 3). Beyond oncology, remodelin has demonstrated therapeutic potential in diverse preclinical disease models. For instance, in cardiac fibrosis, it inhibits NAT10-mediated *BCL-XL* mRNA stabilization to reduce collagen deposition post-myocardial infarction [167]; in vascular remodeling, it suppresses *ITGB1*-FAK signaling to attenuate neointima formation [168]; and in inflammatory bone loss, it destabilizes *Fos* mRNA to block osteoclastogenesis [92]. Additionally, Remodelin alleviates sepsis-induced muscle atrophy by downregulating ROS/NLRP3 pathways [169], mitigates MASLD/MASH by suppressing hepatic *Srebp-1c*-driven lipogenesis [85], and improves rheumatoid arthritis by destabilizing *PTX3* mRNA to reduce synovial aggression [79]. Its efficacy extends to heart regeneration impairment [78], ischemia–reperfusion injury [75], HIV-1 replication inhibition [170], and obesity [171] through modulation of mitochondrial respiration, ferroptosis, viral RNA stability, and adipogenesis pathways, respectively. These findings underscore Remodelin’s versatility as a NAT10-targeted therapy across both neoplastic and non-neoplastic diseases.

**Table 3:** NAT10 inhibitors and the combination therapies in diseases

NAT10 inhibitor	Diseases	Functions and mechanisms	Combination therapies	Ref
Remodelin	HGPS	Remodelin mediates nuclear shape rescue in laminopathic cells via microtubule reorganization	**-**	[20]
Remodelin	Cervical cancer	Remodelin profoundly improved the efficacy of PD-L1 blockade therapy by impairing tumor cell glycolysis, reducing lactic acid production, and enhancing immunosurveillance within the TME, ultimately resulting in tumor regression	Combination of Remodelin and PD-L1 blockade	[135]
Remodelin	Gastric cancer	Remodelin exhibits anti-gastric cancer activity and enhances the sensitivity of p53 wildtype gastric cancer to MDM2 inhibitors	Combination of Remodelin and MDM2 inhibitors	[110]
Remodelin	Colon cancer	Remodelin inhibits colon cancer cell stemness properties and enhances chemosensitivity of colon cancer by inhibiting the NAT10/NANOGP8 axis	Combination of Remodelin and Oxaliplatin (L-OHP) or Irinotecan (CPT-11)	[174]
Remodelin	Triple-negative breast cancer	Remodelin elevates the surface expression of CTLA-4 on T cells. The combination of remodelin and CTLA-4 mAb can further activate T cells and inhibite tumor progression	Combination of Remodelin and CTLA-4 mAb	[106]
Panobinostat	Hepatocellular carcinoma	Panobinostat binds to NAT10’s catalytic pocket, suppressing its ac4C acetyltransferase activity and attenuating HCC progression	-	[175]
Remodelin	Breast cancer	Remodelin inhibits NAT10 and enhances the sensitivity of breast cancer cells to DNA-damaging agents	Combination of Remodelin and DNA-damaging chemotherapy or radiotherapy	[72]
Remodelin	Head and Neck Squamous Cell Carcinoma	Remodelin inhibits HNSCC tumorigenesis and remodels the tumor microenvironment by increasing infiltrated CD8 + T cells and impairing angiogenesis and Treg recruitment	Combination of Remodelin and immunotherapy	[99]
Remodelin	Melanoma	Remodelin suppresses melanogenesis by repressing MITF (Microphthalmia-Associated Transcription Factor) and its melanogenic targets	-	[134]
Remodelin	Various cancer types	Remodelin enhances the antitumor effects of immunotherapy by regulating PD-L1 expression	Combination of Remodelin and an anti-CTLA-4 antibody	[107]
Remodelin	Multiple myeloma	Remodelin suppreses MM cell proliferation, induces cell cycle arrest and apoptosis in vitro and improves the survival of MM mice in vivo	-	[132]
Remodelin	Bladder cancer	Remodelin increases bladder cancer cisplatin sensitivity by disrupting NAT10-mediated gene regulation through directly inhibiting its expression	Combination of Remodelin and cisplatin	[47]
Remodelin	Nasopharyngeal carcinoma	Remodelin significantly inhibits SLC7 A11 expression and promotes sorafenib-induced ferroptosis in NPC cells	Combination of Remodelin and sorafenib	[109]
Remodelin	Gastric cancer	Remodelin promotes cancer cell escape from glycolytic dependence, thereby potentiating apatinib's antitumor effects	Combination of Remodelin and apatinib	[46]
paliperidone and AG-401	Osteosarcoma	Paliperidone and AG-401 suppress osteosarcoma progression via the NAT10/ac4C/ATF4/ASNS/Asn axis	Combination of paliperidone and AG-401	[162]
Remodelin	Cardiac fibrosis	Remodelin inhibits NAT10-mediated BCL-XL mRNA stabilization, which reduces collagen deposition post-myocardial infarction	-	[167]
Remodelin	Vascular remodeling	Remodelin suppresses ITGB1-FAK signaling to attenuate neointima formation	-	[168]
Remodelin	Inflammatory bone loss	Remodelin destabilizes Fos mRNA to block osteoclastogenesis	-	[92]
Remodelin	Sepsis	Remodelin alleviates sepsis-induced muscle atrophy by downregulating ROS/NLRP3 pathways	-	[169]
Remodelin	MASLD/MASH	Remodelin mitigates MASLD/MASH by suppressing hepatic Srebp-1c-driven lipogenesis	-	[85]
Remodelin	Rheumatoid arthritis	Remodelin improves rheumatoid arthritis by destabilizing PTX3 mRNA to reduce synovial aggression	-	[79]
Remodelin	Heart regeneration	Remodelin inhibits heart regeneration by relieving the suppression of NAT 10 on the expression of Uqcr11 and Uqcrb, improving mitochondrial respiration and alleviates the glycolytic capacity of the cardiomyocytes	-	[78]
Remodelin	ischemia–reperfusion injury	Remodelin inhibits NAT10-mediated cardiomyocyte ferroptosis, which relieving cardiac ischemia‒reperfusion (I/R) injury	-	[75]
Remodelin	HIV-1 infection	Remodelin inhibits HIV-1 replication by reducing viral RNA stability	-	[170]
Remodelin	obesity	Remodelin reduces body weight, adipocyte size, and adipose tissue expansion in high-fat diet-fed mice by inhibiting KLF9 mRNA ac4C modification	-	[171]
Fosaprepitant	Cancer	Fosaprepitant interacts with NAT10 amino residues ARG725, showing high stability. Inhibition of NAT10 leads to elevated DNA damage, reduced cell survival, and cell cycle arrest	Combination of Fosaprepitant and the standard treatment regimen	[172]
Fludarabine	Cancer	Fludarabine interacts with NAT10 amino residues ARG725, showing high stability. Inhibition of NAT10 leads to elevated DNA damage, reduced cell survival, and cell cycle arrest	Combination of Fludarabine and the standard treatment regimen	[172]
Dantrolene	Cancer	Dantrolene interacts with NAT10 amino residues SER642 and have better binding capability when compared with Remodelin. Inhibition of NAT10 leads to elevated DNA damage, reduced cell survival, and cell cycle arrest	Combination of Dantrolene and the standard treatment regimen	[172]
Folinic acid	Cancer	Folinic acid binds to the SER642 amino acid residue of NAT10 and exhibits higher binding affinity than Remodelin. Inhibition of NAT10 leads to elevated DNA damage, reduced cell survival, and cell cycle arrest	Combination of Folinic acid and the standard treatment regimen	[172]

Besides remodelin, several drugs, including fosaprepitant, folinic acid, fludarabine, dantrolene, paliperidone and AG-401 are also selected as potential NAT10 inhibitors [162, 172]. These inhibitors exhibit higher binding affinity and lower IC values, suggesting superior inhibitory potency compared to remodelin. Notably, fosaprepitant, folinic acid, fludarabine, and dantrolene are FDA-approved drugs with diverse clinical applications, highlighting their potential for drug repurposing, while paliperidone and AG-401 demonstrate synergistic effects in combination therapy, further expanding their therapeutic utility against NAT10-driven malignancies.

Despite promising preclinical evidence supporting the translational potential of NAT10 inhibitors, several critical challenges hinder their clinical advancement. First, limited drug selectivity remains a major concern due to NAT10’s involvement in essential biological processes, such as ribosomal RNA modification. Prolonged inhibition may inadvertently disrupt normal cellular functions, including telomere maintenance, thereby compromising tissue homeostasis. Second, the lack of standardized methodologies to detect ac4C modifications creates barriers to identifying patient populations most likely to benefit and objectively evaluating therapeutic outcomes [32, 173]. Third, optimizing drug delivery systems—for instance, through nanoparticle-based strategies—is imperative to enhance tumor-targeted accumulation and reduce systemic toxicity. Addressing these challenges is essential to unlocking the full therapeutic potential of NAT10 inhibitors.

### Gene silencing approaches

Research demonstrates that targeted suppression of NAT10 expression via CRISPR/Cas9 or short hairpin RNA (shRNA) significantly disrupts its mediated ac4C RNA modifications and associated pathological processes. For instance, in obesity models, NAT10 knockdown reduces ac4C modification on KLF9 mRNA, destabilizes its transcripts, and suppresses downstream CEBPA/B-PPARG pathway activity, thereby inhibiting adipogenesis and alleviating high-fat diet-induced obesity [171]. In acute myeloid leukemia (AML), NAT10 knockout blocks the translational efficiency of target genes such as SLC1 A4 and HOXA9/MENIN, disrupts serine metabolic reprogramming, impairs leukemia stem cell self-renewal, and enhances chemotherapeutic sensitivity [176]. Furthermore, in metabolic dysfunction-associated steatotic liver disease (MASLD), silencing NAT10 markedly reduces hepatic lipogenesis markers and improves hepatic steatosis [177].

In summary, genetic or pharmacological targeting of NAT10 provides critical experimental insights for developing therapeutic strategies in these fields.

## Challenges and future directions in NAT10 research

Ac4C modifications of RNAs are increasingly recognized for their roles in human diseases, such as cancer, neurological diseases, cardiovascular diseases and et al. In the present review, we comprehensively summarize the discoveries, physiological roles and mechanisms of NAT10 in diseases which is the only writer of ac4C modification.

The study field of NAT10 in ac4C modifications is rapidly evolving with ongoing research and discoveries. Identifying the ac4C modification and the target proteins of NAT10 requires innovative technologies that can accurately detect and characterize. Advanced techniques, such as liquid chromatography-mass spectrometry (LC–MS), high-performance liquid chromatography-mass spectrometry (HPLC–MS) and RNA immunoprecipitation-LC/HPLC–MS have been instrumental in discovering and detecting ac4C RNA modifications [10, 102]. Antibody-based methods, such as ac4C antibody pull-down or immunoprecipitation, can selectively enrich ac4C modifications [178–180]. Next-generation sequencing technologies, such as acRIP-seq has been developed to specifically capture and profile ac4C modifications [46, 93]. Advancements in artificial intelligence (AI) and machine learning (ML) have made the progression of RNA modification take a great leap forward [181, 182]. AI and ML algorithms are capable of analyzing large-scale sequencing data to predict and identify new RNA modifications [183, 184]. They do this by learning the patterns and signatures related to known modifications and then using this knowledge to detect and classify novel ones [185]. Along with the progress in data analysis and bioinformatics tools, these technologies have made a great contribution to the identification and characterization of known RNA modifications.

Concerning the mechanism of NAT10 in regulating ac4C modification, it should be noted that ac4C modifications do not work independently, rather, they often interact with and affect one another [186]. NAT10 needs the assistance of THUMPD1 and snoRNA to catalyze ac4C in tRNA and 18 rRNA. respectively. However, it is unclear whether NAT10 requires a cofactor in mRNA ac4C modification. m6 A modification is a reversible and dynamic process in mammalian cells, where it can be written by m6 A methyltransferases (“writers”) and erased by m6 A demethylases (“erasers”) [187, 188]. Whether there are other “writer” and “eraser” factors taking part in the ac4C modification is unclear [27]. Further studies investigating the interaction and cross-talk among NAT10 and other regulators in tRNA, rRNA and mRNA ac4C modifications will offer understanding of mechanisms in ac4C modification regulation network.

In this review, we discussed the translocation of NAT10 in cellular function. Normally, NAT10 is predominantly distributed in the nucleolus. NAT10 is involved in fundamental transcriptional regulation processes. In the nucleolus, NAT10 is an essential regulator of cellular plasticity and plays an important role in chromatin signaling. It can mediate ac4C modifications of proteins which are fate-instructive chromatin regulators. For example, by ac4C modification of the histone chaperone ANP32B, NAT10 can modulate the chromatin landscape of their downstream genes [189]. Under some circumstances, NAT10 could translocate from the nucleolus to the nucleus. In the nucleus, NAT10 is involved in post-transcriptional regulation. It could interact with mRNA molecules and regulate their stability, translation, or localization. For example, DNA damage induced increased localization of NAT10 in the midbody zone of nucleus. The midbody is a structural organelle formed in late phase mitosis which is responsible for completion of cytokinesis. NAT10-mediated acetylation of α-tubulin enhanced the stability of α-tubulin and played an important role in cell division. The depletion of NAT10 induced defects in nucleolar assembly, cytokinesis and G2/M cell cycle arrest or delay of mitotic exit [36]. For another example, upon chemotherapy, NAT10 translocates from the nucleolus to the nucleus to bind with ACLY and acetylates ACLY at K468 to stabilize ACLY and provide drug resistance upon chemotherapy [74]. Therefore, these studies indicated that the distinct subcellular localization of NAT10 has distinct functions. This dynamic subcellular relocation underscores the versatile roles of NAT10 in cellular responses to genotoxic stress, highlighting its significance as a regulatory node in the complex network governing cell fate under different cellular conditions.

Taken together, by scrutinizing the intricate mechanisms of NAT10-mediated ac4C modifications within cellular processes, researchers are uncovering hidden links between ac4C modification and cancer. This comprehensive approach not only aids in the identification of biomarkers for earlier cancer detection but also illuminates targets for therapeutic exploitation. As our understanding of ac4C modification continues to deepen, it may lead to new breakthroughs in the diagnosis, treatment, and prevention of cancers.

## Data Availability

Not applicable.

## Abbreviations

ac4 C: N^4^-acetylcytidine
BLCA: Bladder urothelial carcinoma
CC: Cervical cancer
CoA: Coenzyme A
CRC: Colorectal cancer
ESCA: Esophageal cancer
FSP1: Ferroptosis suppressor protein 1
GC: Gastric cancer
GNAT: Gcn5-related N-acetyltransferase
HCC: Hepatocellular carcinoma
KAT: Lysine acetyltransferases
lncRNA: Long noncoding RNA
m5 C: 5-Methylcytosine
m6 A: N6-methyladenosine
m7G: 7-Methylguanosine
MM: Multiple myeloma
MORC2: MORC family CW-type zinc finger 2
NAT10: N-acetyltransferase 10
OSCC: Oral squamous cell carcinoma
PARP1: Poly (ADP-ribose) polymerase 1
PDAC: Pancreatic ductal adenocarcinoma
PTBP1: Polypyrimidine tract-binding protein 1
PTM: Posttranslational modifications
rRNA: Ribosomal RNA
snoRNA: Small nucleolar RNA
tRNA: Transfer RNA
CDS: CDP-Diacylglycerol Synthase 1
TmcA: TRNA(Met) cytidine acetyltransferase TmcA
E.Coli: Escherichia coli
ANP32B: Acidic Nuclear Phosphoprotein 32 Family Member B
ACLY: ATP Citrate Lyase
Tfec: Transcription factor EC
Uqcr11: Ubiquinol-Cytochrome C Reductase, Complex III Subunit XI
Uqcrb: Ubiquinol-Cytochrome C Reductase Binding Protein
USP18: Ubiquitin Specific Peptidase 18
GPX1: Glutathione Peroxidase 1
RGL1: Ral Guanine Nucleotide Dissociation Stimulator Like 1
RACK1: Receptor For Activated C Kinase 1
TFRC: Transferrin Receptor
FATP2: Fatty acid transport protein 2
EGFR: Epidermal Growth Factor Receptor
SOX4: SRY-Box Transcription Factor 4
AKT1: AKT Serine/Threonine Kinase 1
KIF23: Kinesin Family Member 23
HMGA1: High Mobility Group AT-Hook 1
HMGB2: High Mobility Group Box 2
SRSF2: Serine And Arginine Rich Splicing Factor 2
MMP1: Matrix Metallopeptidase 1
ANKZF1: Ankyrin Repeat And Zinc Finger Peptidyl TRNA Hydrolase 1
CCL2: C–C Motif Chemokine Ligand 2
MAST1: Microtubule Associated Serine/Threonine Kinase 1
KIF23: Kinesin Family Member 23
Wnt/β-catenin pathway: Wnt Beta Catenin pathway
eEF2: Eukaryotic Translation Elongation Factor 2
YTHDF1: YTH N6-Methyladenosine RNA Binding Protein F1
YAP1: Yes1 Associated Transcriptional Regulator
CCL2: C–C Motif Chemokine Ligand 2
COL5 A1: Collagen Type V Alpha 1 Chain
MAPK/ERK: Mitogen-Activated Protein Kinase
SIRT1: Sirtuin 1
ULK1: Unc-51 Like Autophagy Activating Kinase 1
MDM2: MDM2 Proto-Oncogene
MITF: Melanocyte Inducing Transcription Factor
ELOVL6: ELOVL Fatty Acid Elongase 6
ACSL1: Acyl-CoA Synthetase Long Chain Family Member 1
ACSL3: Acyl-CoA Synthetase Long Chain Family Member 3
ACSL4: Acyl-CoA Synthetase Long Chain Family Member 4
ACADSB: Acyl-CoA Dehydrogenase Short/Branched Chain
ACAT1: Acetyl-CoA Acetyltransferase 1
ACOT7: Acyl-CoA Thioesterase 7
LDHA: Lactate Dehydrogenase A
SEPT9: SEPTIN9
YTHDC1: YTH N6-Methyladenosine RNA Binding Protein C1
JunB: JunB Proto-Oncogene, AP-1 Transcription Factor Subunit
HIF-1: Hypoxia Inducible Factor 1
PFKM: Phosphofructokinase, Muscle
PD-1: Programmed cell death protein 1
GAS5: Growth Arrest Specific 5
CXCL10: C-X-C Motif Chemokine Ligand 10
CCL5: C–C Motif Chemokine Ligand 5
FOXP1: Forkhead Box P1
GLUT4: Glucose transporter type 4
KHK: Ketohexokinase
PD-L1: Programmed death-ligand 1
MORC2: MORC Family CW-Type Zinc Finger 2
ACLY: ATP Citrate Lyase
HSP90 AA1: Heat Shock Protein 90 Alpha Family Class A Member 1
AHNAK: AHNAK Nucleoprotein
NF-κB: Nuclear factor-κ B
HOXC8: Homeobox C8
FOXP1: Forkhead Box P1
UTR: Untranslated region
SIRT1: Sirtuin 1
Che-1: Transcription factor che-1
GSK-3β: Glycogen Synthase Kinase 3 Beta
PARP1: Poly(ADP-Ribose) Polymerase 1
PARylation: Poly ADP-ribosylation
IGF2BP1: Insulin Like Growth Factor 2 MRNA Binding Protein 1
ACOT7: Acyl-CoA Thioesterase 7
NOTCH3: Notch Receptor 3
LMNA: Lamin A/C
MDM2: MDM2 Proto-Oncogene
CTLA-4: Cytotoxic T-Lymphocyte Associated Protein 4
acRIP-seq: Acetylated RNA immunoprecipitation and sequencing
THUMPD1: THUMP Domain Containing 1
YWHAE: Tyrosine 3-monooxygenase/tryptophan 5-monooxygenase activation protein epsilon
